# Nodal T Follicular Helper Cell Lymphoma With Epstein–Barr Virus‐Positive Neoplastic T Cells Derived From Clonal Hematopoiesis

**DOI:** 10.1002/jha2.70449

**Published:** 2026-10-28

**Authors:** Daichi Kishi, Nobuhiro Hiramoto, Daisuke Yamashita, Hayato Maruoka, Yumi Shiroishi, Masashi Nishikubo, Yoshimitsu Shimomura, Ryusuke Yamamoto, Yuya Nagai, Yuka Torii, Hiroshi Kimura, Yasuhito Nannya, Seishi Ogawa, Tadakazu Kondo

**Affiliations:** ^1^ Department of Hematology Kobe City Medical Center General Hospital Kobe Japan; ^2^ Department of Pathology Kobe City Medical Center General Hospital Kobe Japan; ^3^ Department of Clinical Laboratory Kobe City Medical Center General Hospital Kobe Japan; ^4^ Department of Pediatrics Nagoya University Graduate School of Medicine Nagoya Japan; ^5^ Department of Virology Nagoya University Graduate School of Medicine Nagoya Japan; ^6^ Department of Pathology and Tumor Biology Kyoto University Kyoto Japan; ^7^ Division of Hematopoietic Disease Control Institute of Medical Science The University of Tokyo Tokyo Japan; ^8^ Institute For the Advanced Study of Human Biology (WPI‐ASHBi) Kyoto University Kyoto Japan

**Keywords:** clonal hematopoiesis, Epstein–Barr virus, nodal T follicular helper cell lymphoma, RHOA, TET2

## Abstract

**Introduction:**

Nodal T follicular helper cell lymphoma (nTFHL) with Epstein–Barr virus (EBV)‐positive neoplastic T cells is rare.

**Methods:**

We report a woman with nTFHL and analyzed EBV‐infected cells using an immunobead‐based method and assessed somatic mutations in lymphoma and lymphoma‐uninvolved bone marrow by targeted sequencing.

**Results:**

EBV DNA was enriched in the CD4‐positive fraction containing neoplastic T cells. Shared *TET2* mutation was identified in both lymphoma and bone marrow, whereas additional *TET2* and *RHOA G17V* mutations were restricted to the lymphoma.

**Conclusion:**

These findings support a stepwise model of EBV‐positive nTFHL arising from *TET2*‐mutated clonal hematopoiesis with subsequent acquisition of a *RHOA* mutation.

**Trial Registration:**

The authors have confirmed clinical trial registration is not needed for this submission.

## 1 Introduction

Nodal T follicular helper cell (TFH) lymphomas (nTFHLs) are neoplasms of mature T cells that express TFH markers such as PD1, ICOS, BCL6, CXCL13, and CD10 [1, 2]. Each nTFHL is classified into one of three categories according to its morphology: nTFHL, angioimmunoblastic type (nTFHL‐AI); nTFHL, follicular type; and nTFHL, not otherwise specified. The lymphomagenesis of nTFHLs is largely driven by the stepwise acquisition of somatic gene mutations [3]. First, *TET2* and *DNMT3A* mutations occur early in hematopoietic stem cells, a process referred to as clonal hematopoiesis. Subsequently, TFH cells acquire an additional *RHOA* mutation, which results in enhanced T‑cell receptor signaling. Epstein–Barr virus (EBV)‐positive B cells, detected by in situ hybridization for EBV‑encoded small RNA (EBER), are present in approximately 70% of nTFHL cases, whereas the neoplastic TFH cells are, as a rule, EBER‑negative [4]. However, to date, 13 cases of nTFHL harboring EBV‑positive neoplastic TFH cells have been reported [5, 6, 7]. nTFHLs with EBV‑positive neoplastic cells remain extremely rare, and their clinical and genetic features as well as their prognosis have yet to be fully elucidated.

The present report describes a patient diagnosed with nTFHL‐AI with EBV‐positive neoplastic T cells. We analyzed subcutaneous mass and lymph node samples involved by lymphoma, and a bone marrow sample without evidence of lymphoma using targeted next‐generation sequencing (NGS). All three samples harbored a common *TET2* mutation, whereas only the lymphoma samples revealed an additional *RHOA* mutation, suggesting that the lymphoma originated from clonal hematopoiesis. The lymphoma was highly aggressive and refractory to chemotherapy. Nevertheless, the patient underwent allogeneic hematopoietic stem cell transplantation and remains in remission two years after transplantation.

## 2 Case Presentation

A 55‐year‐old woman was referred to our hospital with a persistent fever, generalized rash, and multiple subcutaneous masses that had gradually progressed over the previous 3 months. She had no significant past medical history and no family history of malignancy. An ^18^F‐fluorodeoxyglucose positron emission tomography/computed tomography (18F‐FDG PET/CT) scan showed FDG uptake in the cervical, supraclavicular, axillary, para‐aortic, iliac, and inguinal lymph nodes, spleen, and multiple subcutaneous masses. The complete blood count revealed a white blood cell count of 4600/µL with 51% eosinophils, a hemoglobin level of 9.8 g/dL with a normal mean corpuscular volume, and a platelet count of 37,000/µL. Liver and renal function test results were within normal limits. Additional laboratory tests showed a lactate dehydrogenase level of 395 U/L, a β2‐microglobulin level of 3.8 mg/L, and a soluble interleukin‐2 receptor level of 8889 U/mL. Serum immunofixation electrophoresis identified an IgG‐κ monoclonal protein (serum IgG, 1054 mg/dL). The viral capsid antigen (VCA)‐IgG, VCA‐IgM, and Epstein–Barr nuclear antigen (EBNA)‐IgG titers were 160‐fold, < 10‐fold, and 20‐fold, respectively. The EBV genome load in whole blood was 4.87 × 10^6^ IU/mL. Southern blot analysis of EBV terminal repeat regions showed a monoclonal pattern. Serologic testing for human T‐cell leukemia virus type 1 was negative. The lymph node (Figure 1) and subcutaneous mass (Figure S1) biopsy specimens had dense clusters of small‐ to medium‐sized atypical lymphoid cells with prominent high endothelial venules. Immunohistochemical staining of the lymphoma cells was positive for CD3, CD4, CD5, CD10, BCL6, PD1, and ICOS, and negative for CD8 and CD20. Cytotoxic molecules such as perforin, TIA1, and granzyme B were also negative, whereas these markers were partially positive in reactive CD8‐positive small lymphocytes. On serial sections, EBER‐positive cells showed a distribution closely corresponding to that of the CD3‐, CD4‐, and PD1‐positive atypical lymphoid cells and distinct from that of CD20‐positive B cells (Figure 2, Figure S2). At high magnification, EBER‐positive nuclei were observed predominantly within areas composed of morphologically atypical small‐ to medium‐sized lymphoid cells. Multicolor flow cytometry showed that the neoplastic T‐cell population was positive for CD2, CD4, CD5, CD10, ICOS, PD1, and HLA‐DR, and negative for surface CD3, CD7, CD8, CD19, CD20, and CD56. Bone marrow smear morphology and biopsy histopathology, including immunohistochemistry, showed no evidence of lymphoma involvement. Multicolor flow cytometry of the bone marrow sample also revealed no lymphoma cell involvement (< 0.1% of total mononuclear cells). T‐cell receptor (TCR) gene rearrangement was positive for TCRβ in both the lymph node and subcutaneous mass, providing molecular support for T‐cell clonality. In contrast, TCR rearrangement was negative in the bone marrow. Immunoglobulin heavy‐ and light‐chain gene rearrangements were negative in all three specimens. These results confirmed the diagnosis of nTFHL‐AI with EBV‐positive neoplastic T cells. The patient received one cycle of CHOP therapy (cyclophosphamide, doxorubicin hydrochloride, vincristine sulfate, and prednisone), but the lymphoma was refractory to chemotherapy and progressed to a leukemic phase. After achieving a partial remission with dose‐adjusted EPOCH therapy (etoposide, prednisone, vincristine sulfate, cyclophosphamide, and doxorubicin hydrochloride), she underwent allogeneic hematopoietic stem cell transplantation from her HLA‐haploidentical son. Two years after transplantation, she remains alive and in complete remission.

**FIGURE 1:**
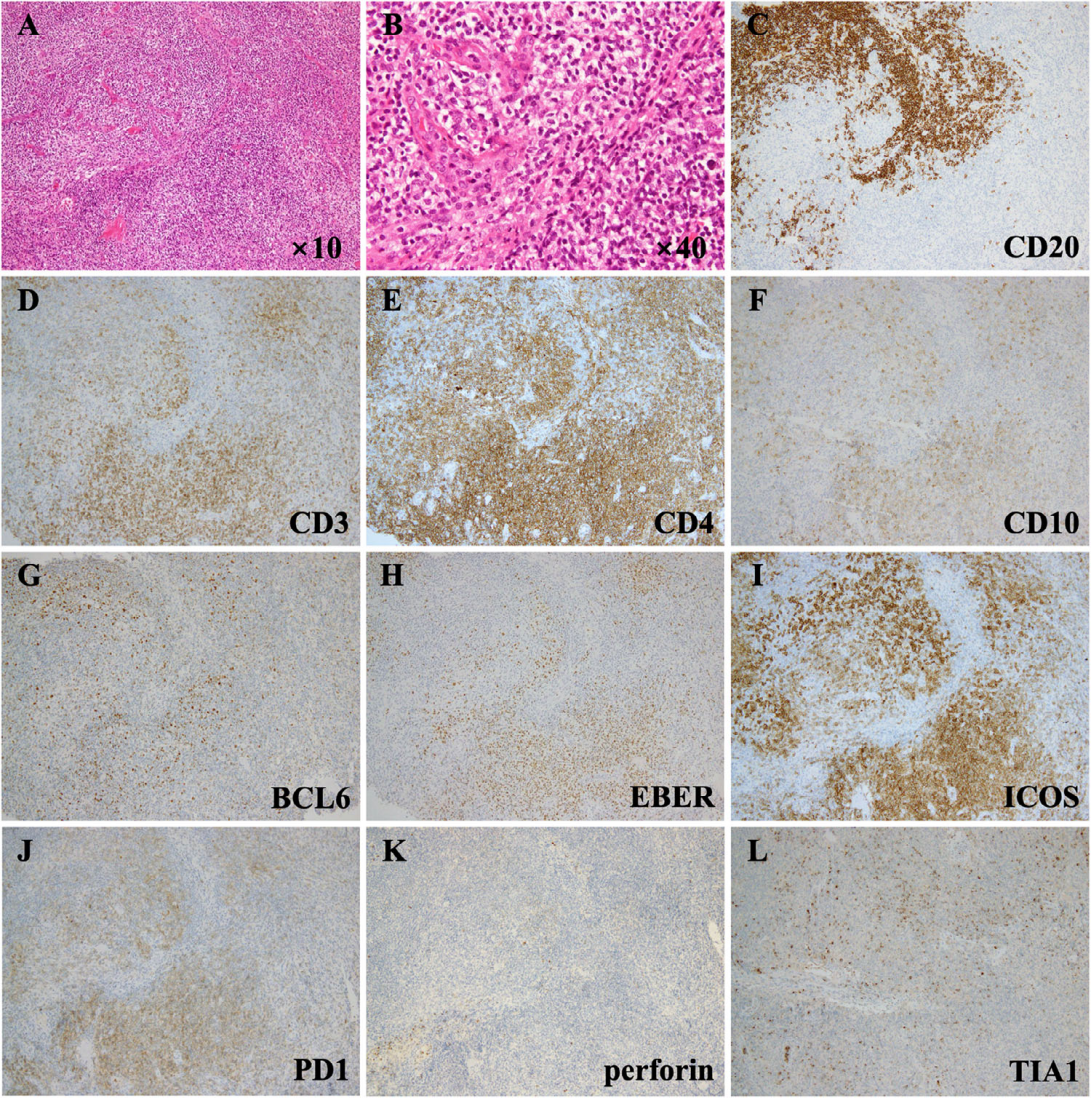
Histopathology of the lymph node. (A, B) Hematoxylin and eosin (A: × 10, B: × 40); (C) CD20; (D) CD3; (E) CD4; (F) CD10; (G) BCL6; (H) EBER; (I) ICOS; (J) PD1; (K) perforin; (L) TIA1.

**FIGURE 2:**
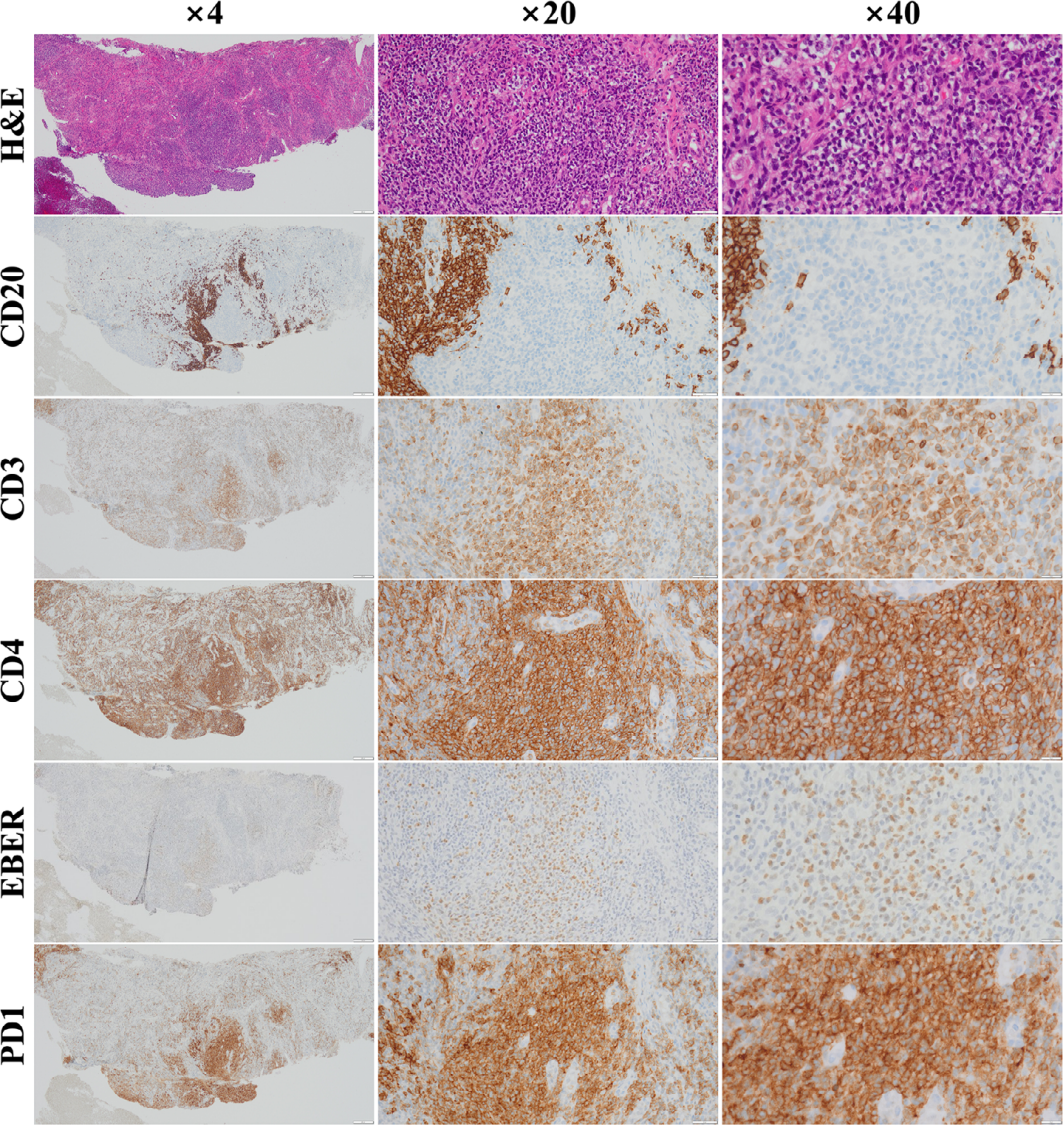
Histopathological distribution of EBER‐positive cells in the lymph node. Representative serial sections stained with hematoxylin and eosin (H&E), CD20, CD3, CD4, EBER, and PD1 at low (×4), intermediate (×20), and high (×40) magnification. EBER‐positive cells showed a distribution corresponding to the CD3‐, CD4‐, and PD1‐positive atypical lymphoid cell population and distinct from that of CD20‐positive B cells. At high magnification, EBER‐positive nuclei were observed within areas composed predominantly of morphologically atypical lymphoid cells.

We conducted two studies. First, we determined which blood cell populations harbored EBV by applying real‐time quantitative polymerase chain reaction analysis for EBV DNA to blood cells obtained during the leukemic phase that had been sorted using an immunobead‐based method [8]. Second, we determined whether the stepwise acquisition of somatic gene mutations occurs in the lymphoma by comparing mutational profiles between subcutaneous mass and lymph node samples involved by lymphoma and a bone marrow sample without evidence of lymphoma using targeted NGS. The present study was approved by the Ethics Committee of Kobe City Medical Center General Hospital, and written informed consent was obtained from the patient.

In the initial analysis, peripheral blood mononuclear cells were fractionated into CD3‐positive, CD4‐positive, CD8‐positive, CD19‐positive, and CD56‐positive populations using an immunobead‐based method. Each fraction was then examined for EBV DNA using real‐time quantitative polymerase chain reaction analysis. Flow cytometry performed on the same peripheral blood sample used for the immunobead analysis showed that the CD3‐negative/CD4‐positive neoplastic population accounted for 5.93% of total analyzed cells. The CD4‐positive fraction, which contained the neoplastic T cells, showed the highest EBV DNA level (789,790 IU/µg DNA) among the examined cell populations (Table S1). A substantial EBV DNA level was also detected in the CD19‐positive fraction (290,775 IU/µg DNA), whereas the levels in the CD3‐, CD8‐, and CD56‐positive fractions were lower. Together with the histopathological findings, the enrichment of EBV DNA in the CD4‐positive fraction supports EBV infection of the neoplastic T cells.

In the subsequent analysis, DNA extracted from subcutaneous mass, lymph node, and bone marrow samples was analyzed by targeted NGS covering 140 genes recurrently mutated in lymphoid neoplasms (Table S2). The methods of targeted NGS are described in the Supporting Information. A common *TET2* mutation (NM_001127208: c.2474C>A: p.S825X) was identified in all three samples (Table 1). The subcutaneous mass and lymph node samples involved by the lymphoma harbored additional *TET2* (NM_001127208: c.5397_5418del: p.K1799fs) and *RHOA* (NM_001664: c.50G>T: p.G17V) mutations. The shared *TET2* mutation was also detected in the bone marrow sample without evidence of lymphoma, supporting an origin from pre‐existing clonal hematopoiesis. No other pathogenic variants were identified in any of the three samples.

**TABLE 1:** Pathogenic variants identified by targeted next‐generation sequencing.

Gene	NM_ID	Nucleotide change	Amino acid change	nTFHL(SM) VAF	nTFHL(LN) VAF	BM VAF
**Common for nTFHL and bone marrow**						
*TET2*	NM_001127208	c.2474C > A	p.S825X	0.378	0.306	0.409
**Specific for bone marrow**						
None detected	N.A.	N.A.	N.A.	N.A.	N.A.	N.A.
**Specific for nTFHL**						
*RHOA*	NM_001664	c.50G>T	p.G17V	0.269	0.128	0
*TET2*	NM_001127208	c.5397_5418del	p.K1799fs	0.168	0.129	0

Abbreviations: BM, bone marrow; LN, lymph node; N.A., not applicable; nTFHL, nodal T follicular helper cell lymphoma; SM, subcutaneous mass; VAF, variant allele frequency.

## 3 Discussion

Herein, we report a rare case of nTFHL with EBV‐positive neoplastic T cells. Genetic analysis revealed the stepwise acquisition of gene mutations. Although this disease has a poor prognosis, the patient underwent allogeneic stem cell transplantation and achieved long‐term survival. To date, 14 cases, including ours, have been reported as nTFHLs with EBV‑positive neoplastic T cells, which were diagnosed in 7 females and 7 males, aged 55–81 years at diagnosis [5, 6, 7]. Clinical data were available for 11 patients; B symptoms were present in all, generalized lymphadenopathy in 9, and skin lesions in 5. These clinical features appear to be similar to those of nTFHLs in general. Among the 14 reported cases, 13 were classified as nTFHL, including 11 cases of the angioimmunoblastic type, one of the follicular type, and one not otherwise specified. NGS‑based genetic assessment showed that all but one case harbored *RHOA* and *TET2* mutations (Table S3), consistent with the genetic features of nTFHLs. Some nTFHLs develop through a stepwise process in which *TET2* mutations occur early in hematopoietic stem cells, followed by TFH cells acquiring *RHOA* mutations [3]. Although the relationship between these mutations and EBV infection remains unclear, the detection of *TET2* and *RHOA* mutations suggests that nTFHLs with EBV‐positive neoplastic T cells may share a genetic background and a similar pattern of stepwise genetic evolution with conventional nTFHLs. In patient #10 reported by Burroni et al., underlying clonal hematopoiesis was suggested by higher variant allele frequencies (VAFs) of *TET2* and *DNMT3A* mutations than the VAF of a *RHOA* mutation in bulk lymphoma tissue (Table S3) [5]. In contrast, in the present case, the same *TET2* mutation was detected in both lymphoma and lymphoma‐uninvolved bone marrow, whereas the *RHOA* mutation was restricted to the lymphoma samples. Although the use of bulk bone marrow sequencing warrants caution in interpreting the lineage relationship, these findings provide direct support for the presence of clonal hematopoiesis and are consistent with a stepwise model in which the lymphoma subsequently acquired a *RHOA* mutation.

The immunobead analysis showed the highest EBV DNA level in the CD4‐positive fraction, which included the neoplastic cells, further supporting EBV infection of the neoplastic T cells. A substantial EBV DNA level was also detected in the CD19‐positive fraction despite the lack of prominent EBER‐positive B cells in the biopsy specimens, possibly reflecting EBV infection of circulating B cells [9]. Although an IgG‐κ monoclonal protein was detected, immunoglobulin gene rearrangements were negative in all three specimens, and no concomitant B‐cell neoplasm was identified. The relatively low EBV DNA level in the CD3‐positive fraction likely reflects loss of surface CD3 on the neoplastic cells, whereas low‐level signals in the other fractions may partly reflect incomplete fraction purity.

The present case showed both clinical and phenotypic features of nTFHLs and EBV‐positive nodal T‐ and NK‐cell lymphoma; in particular, differentiation from EBV‐positive nodal T‐ and NK‐cell lymphoma was crucial. EBV‐positive nodal T‐ and NK‐cell lymphoma also has an aggressive clinical course and may be associated with clonal hematopoiesis harboring *TET2* mutations [6]. In contrast, the presence of a *RHOA* mutation and the absence of cytotoxic molecule expression in the present case helped exclude the diagnosis of EBV‐positive nodal T‐ and NK‐cell lymphoma. In addition, as a characteristic finding of EBV‑positive nodal T‑ and NK‑cell lymphoma, the EBV‑positive neoplastic T cells are almost constantly negative for CD5, whereas CD5 expression was retained in the present case [10]. These findings may help differentiate between the two entities.

Thus far, 10 patients with available follow‑up data, including our case, received chemotherapy; among them, one patient underwent allogeneic stem cell transplantation and another underwent autologous stem cell transplantation [5, 6, 7]. All patients except the present case died, experiencing rapid disease progression and succumbing 2–11 months after diagnosis. This disease carries an extremely poor prognosis, and durable remission is unlikely with chemotherapy alone. In patients who can tolerate allogeneic hematopoietic stem cell transplantation, such treatment may therefore be considered. Further accumulation of similar cases is warranted to facilitate elucidation of the disease pathogenesis, refinement of diagnostic criteria, and optimization of treatment strategies for this rare entity.

## Author Contributions

Daichi Kishi and Nobuhiro Hiramoto designed the case report. Daisuke Yamashita contributed to the histological diagnosis. Yasuhito Nannya performed the sequencing data analyses. Hayato Maruoka and Yumi Shiroishi conducted multicolor flow cytometry analyses. Hiroshi Kimura and Yuka Torii identified Epstein–Barr virus (EBV)‐infected blood cells in peripheral blood samples. Daichi Kishi, Yoshimitsu Shimomura, Yuya Nagai, and Nobuhiro Hiramoto managed the patient. Nobuhiro Hiramoto and Daichi Kishi wrote the manuscript. All authors participated in the discussion and interpretation of the results and approved the final manuscript.

## Funding

This work was supported by the Ministry of Education, Culture, Sports, Science and Technology (grant number hp160219), and by the Japan Agency for Medical Research and Development (AMED) (grant number JP20cm0106501h0005 to Seishi Ogawa).

## Ethics Statement

The present study was approved by the Ethics Committee of Kobe City Medical Center General Hospital. The study was conducted in accordance with the Declaration of Helsinki.

## Consent

Written informed consent for participation and publication of the clinical details and images was obtained from the patient.

## Conflicts of Interest

The authors declare no conflicts of interest.

## Supporting information




**Supporting Information File 1**: jha270449‐sup‐0001‐Figure S1.docx

**Supporting Information File 2**: jha270449‐sup‐0002‐Figure S1.png

**Supporting Information File 3**: jha270449‐sup‐0003‐Figure S2.docx

**Supporting Information File 4**: jha270449‐sup‐0004‐Figure S2.png

**Supporting Information File 5**: jha270449‐sup‐0005‐SuppMat.docx

**Supporting Information File 6**: jha270449‐sup‐0006‐Table S1.docx

**Supporting Information File 7**: jha270449‐sup‐0007‐Table S2.docx

**Supporting Information File 8**: jha270449‐sup‐0008‐Table S3.docx

## Data Availability

The data are not publicly available owing to privacy or ethical restrictions, but may be available from the corresponding author upon reasonable request and subject to institutional approval.
