# Supplementary material for: Nodal T Follicular Helper Cell Lymphoma With Epstein–Barr Virus‐Positive Neoplastic T Cells Derived From Clonal Hematopoiesis
Source: EJHaem. 2026 Oct 28;7(6):e70449. doi: 10.1002/jha2.70449 (PMC13717066; doi:10.1002/jha2.70449)
Supplement: Supplementary file 1 [file JHA2-7-e70449-s001.docx]

**Supplementary Table 2.** Genes analyzed by targeted next-generation sequencing.

| *ALK* | *CBLB* | *CSNK1A1* | *HLA-A* | *MEF2B* | *PRKCB* | *STAT5B* |
| --- | --- | --- | --- | --- | --- | --- |
| *ARID1A* | *CCND1* | *CSNK2A1* | *HLA-B* | *MYC* | *PRKCQ* | *STAT6* |
| *ARID1B* | *CCND3* | *CSNK2B* | *HLA-C* | *MYD88* | *PTEN* | *SYK* |
| *ARID2* | *CCR4* | *CTCF* | *HNRNPA2B1* | *NCOR1* | *PTPN1* | *TBL1XR1* |
| *ATM* | *CCR7* | *CXCR4* | *ID3* | *NFKB2* | *PTPRC* | *TCF3* |
| *ATP6AP1* | *CD274* | *DDX3X* | *IDH2* | *NFKBIA* | *RB1* | *TET2* |
| *ATP6V1B2* | *CD28* | *DNMT3A* | *IKBKB* | *NFKBIE* | *REL* | *TNFAIP3* |
| *ATXN1* | *CD58* | *DTX1* | *IKZF2* | *NOTCH1* | *RELA* | *TNFRSF14* |
| *ATXN7L1* | *CD70* | *EBF1* | *IRF2BP2* | *NOTCH2* | *RHOA* | *TNFRSF1B* |
| *B2M* | *CD79A* | *EP300* | *IRF4* | *OSBPL10* | *RHOH* | *TP53* |
| *BCL10* | *CD79B* | *EPHA7* | *IRF8* | *PAX5* | *RRAGC* | *TP73* |
| *BCL11A* | *CDK4* | *ETV6* | *ITPKB* | *PDCD1* | *S1PR1* | *TRAF2* |
| *BCL11B* | *CDK6* | *EZH2* | *JAK2* | *PDCD1LG2* | *SETD1B* | *TRAF3* |
| *BCL2* | *CDKN1B* | *FAS* | *JAK3* | *PDE7B* | *SETD2* | *TRAF5* |
| *BCL6* | *CDKN2A* | *FOXO1* | *KDM6A* | *PIK3CD* | *SGK1* | *UBR5* |
| *BIRC3* | *CDKN2B* | *FOXP1* | *KLF2* | *PIK3R1* | *SKI* | *VAV1* |
| *BRAF* | *CDKN2C* | *FYN* | *KLHL14* | *PIM1* | *SMARCA4* | *YTHDF2* |
| *BTG1* | *CEBPA* | *GATA3* | *KMT2C* | *PLCG1* | *SOCS1* | *ZEB1* |
| *BTG2* | *CIITA* | *GNA13* | *KMT2D* | *POT1* | *SPEN* | *ZFP36L2* |
| *CARD11* | *CREBBP* | *GPR183* | *MALT1* | *PRDM1* | *STAT3* | *ZNF638* |
