# Supplementary material for: Nodal T Follicular Helper Cell Lymphoma With Epstein–Barr Virus‐Positive Neoplastic T Cells Derived From Clonal Hematopoiesis
Source: EJHaem. 2026 Oct 28;7(6):e70449. doi: 10.1002/jha2.70449 (PMC13717066; doi:10.1002/jha2.70449)
Supplement: Supplementary file 2 [file JHA2-7-e70449-s002.docx]

**Supplementary Figure 1.** Histopathology of the subcutaneous mass.

A, B, C, Hematoxylin and eosin (A: × 10, B: × 20, C: × 40); D, CD20; E, CD3; F, CD4; G, CD10; H, BCL6; I, EBER; J, ICOS; K, PD1; L, perforin.
