# Supplementary material for: Nodal T Follicular Helper Cell Lymphoma With Epstein–Barr Virus‐Positive Neoplastic T Cells Derived From Clonal Hematopoiesis
Source: EJHaem. 2026 Oct 28;7(6):e70449. doi: 10.1002/jha2.70449 (PMC13717066; doi:10.1002/jha2.70449)
Supplement: Supplementary file 3 [file JHA2-7-e70449-s003.docx]

**Supplementary Figure 2.** Histopathological distribution of EBER-positive cells in the subcutaneous mass. Representative serial sections stained with hematoxylin and eosin (H&E), CD20, CD3, CD4, EBER, and PD1 at low (×4), intermediate (×20), and high (×40) magnification. EBER-positive cells showed a distribution corresponding to the CD3-, CD4-, and PD1-positive atypical lymphoid cell population and distinct from that of CD20-positive B cells. At high magnification, EBER-positive nuclei were observed within areas composed predominantly of morphologically atypical lymphoid cells.
