# Supplementary material for: Nodal T Follicular Helper Cell Lymphoma With Epstein–Barr Virus‐Positive Neoplastic T Cells Derived From Clonal Hematopoiesis
Source: EJHaem. 2026 Oct 28;7(6):e70449. doi: 10.1002/jha2.70449 (PMC13717066; doi:10.1002/jha2.70449)
Supplement: Supplementary file 4 [file JHA2-7-e70449-s004.docx]

**Supplementary Methods**

**Sample collection**

Genomic DNA from the subcutaneous mass, lymph node, and bone marrow of the patient was extracted using the QIAamp DNA Mini kit (Qiagen) and enriched for target regions by liquid-phase hybridization using the SureSelect custom kit (Agilent Technologies), according to the manufacturer’s protocol optimized for automated sample processing, as previously described.^1^ No matched germline normal sample was available. The lymphoma-uninvolved bone marrow sample was analyzed as a comparator but was not considered a germline normal control.

**Targeted sequencing**

The purified library was subjected to high-throughput sequencing analysis on an Illumina HiSeq 2500 or NovaSeq 6000 using the 125 bp or 150 bp paired-end mode. Sequencing reads were aligned to the human genome reference (hg19) using the Burrows–Wheeler Aligner (version 0.7.8) with default parameter settings. Mutation calling was performed through our established pipeline (Genomon pipeline 2.6.0, https://github.com/Genomon-Project), as previously reported.^1-4^ Variants that passed the filtering process were further assessed for oncogenicity using an in-house curation program. The curation policy was determined individually for each gene based on previous reports and databases after excluding variants registered in public SNP databases (the 1000 Genomes Project as of August 2014, ESP6500, Human Genome Variation Database) and call errors using EBCall^5^ and an in-house blacklist of error calls.

The mean target sequencing depth across the subcutaneous mass (SM), lymph node (LN), and bone marrow (BM) samples was 556.8×. The variant allele frequency (VAF) threshold for variant calling was set at ≥2%. At the TET2 p.S825X locus, sequencing depths were 331×, 304×, and 362× in the SM, LN, and BM samples, respectively, with 125, 93, and 148 variant-supporting reads. For TET2 p.K1799fs, sequencing depths were 1,226× and 1,300× in the SM and LN samples, respectively, with 206 and 168 variant-supporting reads. For RHOA p.G17V, sequencing depths were 610× and 548× in the SM and LN samples, respectively, with 164 and 70 variant-supporting reads.

**References**

1. Yoshida K, Sanada M, Shiraishi Y, et al. Frequent pathway mutations of splicing machinery in myelodysplasia. Nature. 2011;478(7367):64-69. Published 2011 Sep 11. doi:10.1038/nature10496
2. Haferlach T, Nagata Y, Grossmann V, et al. Landscape of genetic lesions in 944 patients with myelodysplastic syndromes. Leukemia. 2014;28(2):241-247. doi:10.1038/leu.2013.336
3. Yoshizato T, Dumitriu B, Hosokawa K, et al. Somatic Mutations and Clonal Hematopoiesis in Aplastic Anemia. N Engl J Med. 2015;373(1):35-47. doi:10.1056/NEJMoa1414799
4. Suzuki H, Aoki K, Chiba K, et al. Mutational landscape and clonal architecture in grade II and III gliomas. Nat Genet. 2015;47(5):458-468. doi:10.1038/ng.3273
5. Yoshizato T, Nannya Y, Atsuta Y, et al. Genetic abnormalities in myelodysplasia and secondary acute myeloid leukemia: impact on outcome of stem cell transplantation. Blood. 2017;129(17):2347-2358. doi:10.1182/blood-2016-12-754796
