# Supplementary material for: Nodal T Follicular Helper Cell Lymphoma With Epstein–Barr Virus‐Positive Neoplastic T Cells Derived From Clonal Hematopoiesis
Source: EJHaem. 2026 Oct 28;7(6):e70449. doi: 10.1002/jha2.70449 (PMC13717066; doi:10.1002/jha2.70449)
Supplement: Supplementary file 5 [file JHA2-7-e70449-s006.docx]

**Supplementary Table 3.** Characteristics of reported cases with EBV-positive neoplastic T cells and TFH features.

|  | Age (y)/Sex | Diagnosis | Evidence of EBV-positive neoplastic T cells | Clonal TCR gene rearrangement | Representative genetic mutations (VAF) | | | CH status/evidence | Treatment | Outcome |
| --- | --- | --- | --- | --- | --- | --- | --- | --- | --- | --- |
|  |  |  |  |  | TET2 | DNMT3A | RHOA |  |  |  |
| **Zhang et al. (2023)** |  |  |  |  |  |  |  |  |  |  |
| Case 1 | 59/Male | nTFHL-F | Dual staining (CD3/EBER, CD4/EBER, PD1/EBER), Sanger sequencing in microdissected tumor cells | Detected | p.L748* (31.46%) | Not detected | p.G17V (34.22%) | Not demonstrated | CHOP | DOD, 11 mo |
| Case 2 | 58/Male | nTFHL-AI | EBER-ISH with corresponding morphology/immunophenotype | Detected | p.M1333Rfs*28 (27.59%) | Not detected | p.G17V (28.66%) | Not demonstrated | CHOP | DOD, 8 mo |
| **Kato et al. (2024)** |  |  |  |  |  |  |  |  |  |  |
| UPN 257 | 76/Female | EBV+ nPTCL with nTFHL features | EBER-ISH with corresponding morphology/immunophenotype | NA | p.Q916* (29%) | p.P904L (37%) | p.G17V (29%) | Not demonstrated | No therapy | DOD, 2 mo |
| **Burroni et al. (2025)** |  |  |  |  |  |  |  |  |  |  |
| Patient #1 | 79/Female | nTFHL-AI | Dual staining (CD3/EBER, TFH markers/EBER) | Detected | p.R1404X (35.5%), p.Y620X (32.9%) | Not detected | p.G17V (24.2%) | Not demonstrated | CHOP | DOD, 2 mo |
| Patient #2 | 80/Female | nTFHL-AI | Dual staining (CD3/EBER, TFH markers/EBER) | Detected | p.Q640X (33%), p.R550X (32.5%) | Not detected | p.G17V (27.7%) | Not demonstrated | No data | No data |
| Patient #3 | 70/Male | nTFHL-AI | Dual staining (CD3/EBER, TFH markers/EBER) | Detected | p.I1175fs (40.7%), p.Q731fs (40%) | Not detected | p.G17V (33.6%) | Not demonstrated | CHOP | DOD, 7 mo |
| Patient #4 | 81/Male | nTFHL-NOS | Dual staining (CD3/EBER, TFH markers/EBER) | Detected | p.R1465X (37.5%), p.E796X (33.8%) | Not detected | p.G17V (34.9%) | Not demonstrated | CHOP-like | DOD, 2 mo |
| Patient #5 | 69/Male | nTFHL-AI | Dual staining (CD3/EBER, TFH markers/EBER) | Detected | p.V498fs (40.8%) | p.R635W (22.8%) | NA | Not demonstrated | AVD, pentostatin, ICE, DHAOx, nivolumab | DOD, 6 mo |
| Patient #6 | 67/Female | nTFHL-AI | Dual staining (CD3/EBER, TFH markers/EBER) | Detected | p.Q1414R (20.6%) | p.I780T (21.5%) | p.G17V (19.7%) | Not demonstrated | CHOP,  auto-HSCT, pentostatin | DOD, 5 mo |
| Patient #7 | 77/Female | nTFHL-AI | Dual staining (CD3/EBER) | Not detected | p.H431 (27%), p.Y1345D (27%) | p.Glu240 (29%) | p.G17V (29%) | Not demonstrated | CHOP | DOD, 8 mo |
| Patient #8 | 63/Female | nTFHL-AI | Dual staining (CD3/EBER) | NA | p.Q758X (31.6%) | Not detected | p.G17V (31.5%) | Not demonstrated | No data | No data |
| Patient #9 | 56/Male | nTFHL-AI | Dual staining (CD3/EBER, TFH markers/EBER) | Not interpretable | p.K450fs (29%), p.C1172fs (30%) | p.R326C (27%) | p.G17V (29%) | Not demonstrated | CHOP | DOD, 3 mo |
| Patient #10 | 79/Male | nTFHL-AI | Dual staining (CD3/EBER, TFH markers/EBER) | Detected | c.4044+1G>A (21%) | p.Arg882Cys (33%) | p.D120N (3.8%) | Inferred by higher VAFs of TET2/DNMT3A than RHOA | R-CHOP,  chemotherapy | No data |
| **Present Case** | 55/Female | nTFHL-AI | EBER-ISH with corresponding morphology/immunophenotype | Detected | p.S825X (30.6%), p.K1799fs (12.9%) | Not detected | p.G17V (12.8%) | Supported by the identical TET2 mutation in lymphoma and lymphoma-uninvolved BM; RHOA restricted to lymphoma | CHOP,  DA-EPOCH,  allo-HSCT | Alive in CR, 24 mo after allo-HSCT |

Abbreviations: allo-HSCT, allogeneic hematopoietic stem cell transplantation; auto-HSCT, autologous hematopoietic stem cell transplantation; AVD, doxorubicin, vinblastine, and dacarbazine; BM, bone marrow; CH, clonal hematopoiesis; CHOP, cyclophosphamide, doxorubicin, vincristine, and prednisone; CR, complete remission; DA-EPOCH, dose-adjusted etoposide, prednisone, vincristine, cyclophosphamide, and doxorubicin; DHAOx, dexamethasone, cytarabine, and oxaliplatin; DNMT3A, DNA methyltransferase 3 alpha; DOD, died of disease; EBV, Epstein-Barr virus; EBER, Epstein–Barr virus-encoded small RNA; ICE, ifosfamide, carboplatin, and etoposide; ISH, in situ hybridization; mo, months; NA, not available; nPTCL, nodal peripheral T-cell lymphoma; nTFHL, nodal T follicular helper cell lymphoma; nTFHL-AI, nodal T follicular helper cell lymphoma, angioimmunoblastic type; nTFHL-F, nodal T follicular helper cell lymphoma, follicular type; nTFHL-NOS, nodal T follicular helper cell lymphoma, not otherwise specified; PD1, programmed cell death protein 1; R-CHOP, rituximab plus CHOP; RHOA, Ras homolog family member A; TCR, T-cell receptor; TET2, tet methylcytosine dioxygenase 2; TFH, T follicular helper cell; UPN, unique patient number; VAF, variant allele frequency; y, years.

Note: The timing of EBV infection relative to the acquisition of lymphoma-associated genetic alterations could not be determined in any of the reported cases.
