# Supplementary material for: Nodal T Follicular Helper Cell Lymphoma With Epstein–Barr Virus‐Positive Neoplastic T Cells Derived From Clonal Hematopoiesis
Source: EJHaem. 2026 Oct 28;7(6):e70449. doi: 10.1002/jha2.70449 (PMC13717066; doi:10.1002/jha2.70449)
Supplement: Supplementary file 6 [file JHA2-7-e70449-s008.docx]

**Supplementary Table 1.** EBV DNA levels in immunobead-sorted peripheral blood mononuclear cell fractions.

| Cell fractions | Total DNA  concentration (μg/mL) | EBV DNA (IU/mL) | EBV DNA (IU/µg DNA) |
| --- | --- | --- | --- |
| MNC | 6.5 | 2,512,985 | 386,613 |
| CD3-positive | 1.2 | 26,853 | 22,718 |
| **CD4-positive** | **5.8** | **4,580,782** | **789,790** |
| CD8-positive | 0.5 | 15,288 | 29,976 |
| CD19-positive | 0.2 | 63,389 | 290,775 |
| CD56-positive | 1.1 | 21,862 | 20,547 |

Abbreviations: EBV, Epstein–Barr virus; MNC, mononuclear cells
